# Contributions to Obstetrics, with Tabular Views, and Miscellaneous Practical Observations

**Published:** 1850-10

**Authors:** Henry A. Ramsay

**Affiliations:** Raysville, Georgia


MEDICAL EXAMINER,

AND

RECORD OF MEDICAL SCIENCE.

NEW SERIES.—NO. LXX.—OCTOBER, 1850.

### ORIGINAL COMMUNICATIONS.

*Contributions to Obstetrics, with Tabular Views, and Miscellaneous Practical Observations.* By Henry A. Ramsay, M. D., Raysville, Georgia.

Contributions to obstetrical science from the South are so rare, that I have concluded to present the results of my restricted experience, in this department, in a tabular and commentative form, which, however lame in practical importance, may elicit farther details, and furnish some data for determining the comparative state of rational midwifery in the Southern country. The field of obstetrical observation in the South being confined largely to our black population, presents at many points advantages for the practical elucidation of obstetricy nowhere else to be obtained by the professional man. At the present day a large majority of the obstetrical cases among this class, are exclusively under the control of the physician, and I am happy to add, the white ladies of Georgia are fast falling into ranks, in reference to this important point. Wherever our black population is dense, and their condition healthful, they increase with an almost unparalleled rapidity, and are unusually prolific ; and such is the care with which they are provided for by their owners, to secure these desirable ends, that we hazard nothing in asserting that the negroes of Georgia are better provided for, *obstetrically* and *dietetically*, than any other dependent class upon this continent, *private infirmaries, public hospitals, alms-houses, pauper cliniques, and lying in hospitals*, to the contrary, notwithstanding. The planters have their family physicians, who have the exclusive control of the blacks, and who are called without reference to simple or emergent cases, and without regard to expense. Since my advent into the profession, it has been my good fortune to attend several hundred cases of labor, embracing almost every variety, from the simplest to the most difficult forms; and it has been my better luck not to have sustained a *single loss*. In explanation of this success, it may be remarked that our blacks are well fed, clothed, and favored as to labor, and our white ladies enjoy in an eminent degree, all those luxuries and advantages which conspire to health; they are therefore stout, well formed, and energetic, having seldom any of those pelvic deformities or other causes, which induce laborious or tedious labor; consequently they conceive readily, and bring forth with marked facility. It is true, that we sometimes meet with difficult and fatal labors among both colors, but they are usually dependent upon *too early marriages, accidents*, or other causes beyond the ken of human comprehension or judgment. Such cases will occur among all classes and colors, and in any community. It will be seen by reference to these tables, that puerperal convulsions are quite rare, at least in this section of Georgia, and I apprehend that what is common here is true of other parts of the State. An interesting enquiry presents itself in reference to this point, should it be farther corroborated:—What is the cause of this immunity? Hypothetical disquisition is not the object of this paper, but I would make a suggestive rationale of the causation of this exemption, without fully endorsing or denying its validity—*that it is dependent upon a preponderance of the cerebral over the spinal system.* This is a fruitful and speculative theme, involving many points of acquiescence and contrariety, but I am inclined to think many sound views may be urged in justification of the position, whether the postulate be correct or not. But I will reserve these views for another occasion, and descend to a tabular statement of my cases, appending such remarks as I may deem necessary.

EUTOCIA.

| | |
|---|---|
| *Natural,* | 429 vertex presentations, |
| | 5 twin cases, |
| *Unnatural,* | 3 footlings, |
| | 4 breech, |
| | 1 facial. |
| *Complicated,* | 3 cases arm and shoulder presentation, |
| | 1 case arm, foot and placenta, |
| | 2 cases placenta previa, |
| | 1 case side and funis presenting, |
| | 4 cases puerperal convulsions, |
| | 1 case tumor impeding labor, |
| | 1 case labial effusion, |
| | 2 cases hæmorrhage prior to labor, |
| | 1 case adherent placenta, |
| | 3 cases hour glass contraction, |
| | 1 case cord torn from placenta in utero, |
| | 1 case impacted head. |

DYSTOCIA.

*Manual,*—3 cases required turning.

| | |
|---|---|
| *Instrumental,* | 2 cases required forceps, |
| | 5 cases embryotomy. |

The above table is unavoidably incomplete; in the early part ot my professional career, I carefully recorded all my difficult obstetrical cases, leaving the simple labors to fare for themselves; hence the reader will find an aggregate of 473 cases only, when, in reality, I am confident I have attended six hundred or more. This table exhibits all my recorded cases of every description, ranging from the seventh to the ninth month. It will be seen that I have transcended some obstetricians in the comparative frequency of some forms of difficult labor, but this may find an explanation, (at least in some degree,) in the fact alluded to above, that all the *difficult* labors are recorded, while the simple list is not replete. But amid all this freak of negligence and confusion, I have prepared a tabular numerical and per centage view of my experience, which I am inclined to think will successfully compare with results of some older tokologists, who have a larger experience, and more reputation than myself. If I am correct in this opinion I trust it will give an impetus to Southern Obstetric Medicine, and bring to light many facts of tokological importance, hitherto thrown aside as worthless and unimportant. If this desideratum should be attained, my highest hopes and expectations will be fully consummated.

For the sake of convenience, and the better understanding of the tabular statement, I have arranged the various classes and species of labor in *aggregate and distinctive forms.*

*Aggregate view, No.* 1.

| | | | Frequency. | Per cent. |
|---|---|---|---|---|
| *Natural Labor,* | - - - | 5 cases twins, | 1 in 94,* | or 1.05 |
| *Unnatural Labor,* | - - - | 8 cases, | 1 in 59, | or 1.69 |
| *Complex Labor,* | - - - | 21 cases, | 1 in 22, | or 4.45 |
| *Manual Labor,* | - - - | 3 cases, | 1 in 157, | or 0.63 |
| *Instrument. Labor* | - - - | 7 cases, | 1 in 67, | or 1.06 |

*Distinctive view, No.* 2.

EUTOCIA.

| Presentations. | | No. | Numerical frequency. |
|---|---|---|---|
| Vertex,† | - - - - - - | 429 | 429 in 473 |
| Feet,† | - - - - - - | 3 | 1 in 157 |
| Breech, | - - - - - - | 4 | 1 in 118 |
| Face, | - - - - - - | 1 | 1 in 473 |

DYSTOCIA.

| | Presentation, etc. | No. of cases. | Frequency. |
|---|---|---|---|
| *Complicated, &c.* | Arm and shoulder, - - | 3 | 1 in 157 |
| | Arm, foot and placenta, - | 1 | 1 in 473 |
| | Placenta previa, - - | 2 | 1 in 236 |
| | Side and funis, - - - | 1 | 1 in 473 |
| | Convulsions, - - - | 4 | 1 in 118 |
| | Tumor, - - - - | 1 | 1 in 473 |
| | Labial effusion, - - - | 1 | 1 in 473 |
| | Hemorrhage, - - - | 2 | 1 in 236 |
| | Adherent placenta, - - | 1 | 1 in 473 |
| | Hour glass contraction, - | 3 | 1 in 157 |
| | Cord torn from placenta in utero, | 1 | 1 in 473 |
| | Impacted head, - - - | 1 | 1 in 473 |
| *Manual,* | —Cases required version, - - - | 3 | 1 in 157 |
| *Instrumental,* | Forceps, - - - - - | 2 | 1 in 236 |
| | Required embryotomy or cephalotomy, | 5 | 1 in 94 |

From the aggregate view No. 1, it will be seen that twin cases occur here in the ratio of 1 in 94, or 1.05 per cent. Unnatural labor, including face, feet, and breech presentations occur in the ratio of 1 in 59, or 1.69 per cent. Complex labor, including all *mal-positions, accidental or unforeseen deviations*, occur 1 in 22, or 4.45 per cent. Manual labor, including *turning only*, occurs 1 in 157, or 0.63 per cent., and instrumental labor, including *forceps, embryotomy* and *cephalotomy*, 1 in 67, or 1.06 per cent. It will be remembered that the list of simple eutocia is not

*A general average with Clarke, Boivin and Baudelocque.

†Twin cases not included.

replete; but let us contrast this picture with other authorities, and see how the case stands; and here, permit me to remark, the comparison is instituted with no invidious design, but merely to illustrate the position, and define the standard, as far as compatible, of Southern obstetricy.

*Tabular view of relative frequency of various forms of Labor in French and English Practice.*

| Presentation, &c. | Baudelocque. | Boivin. | Bland.* | Collins. | Merriman. |
|---|---|---|---|---|---|
| Feet, | 1 in 81 | Uncertain | 1 in 105 | 1 in 131 | 1 in 76 |
| Breech, | 1 in 59 | " | 1 in 52 | 1 in 40 | 1 in 00 |
| Face, | 1 in 296 | " | Not known | 1 in 504 | Not rep |
| Arm & shoulder, | 1 in 336 | " | 1 in 210 | 1 in 416 | 1 in 155 |
| Placental, | Not rep. | " | Not rep. | 1 in 1514 | Not rep. |
| Convulsions, | " | " | †1 in 210 | †1 in 693 | 1 in 105† |
| Hemmorrhage, | " | " | Uncertain | †1 in 555 | 1 in 558† |
| Twins, | 1 in 91 | 1 in 132 | Not rep. | 1 in 69 | 1 in 76 |
| Delivered by art, | 1 in 76 | Not rep. | " | Not rep. | Not rep. |

By a brief recapitulation of the preceding tables, it will be seen that the following facts are demonstrated: 1. That we do not have unnatural labors as frequently as the French and English, as far as our tables are capable of determining. 2d. That arm presentations have occurred more frequently to me, than to Baudelocque, Bland, or Collins. 3d. That puerperal convulsions have occurred oftener with me than with Collins or Merriman, although I have previously declared that our ladies enjoy almost an immunity, and attempted to account for it; this unique position will find some atonement by recurring to Dr. Bland's experience in 1897 cases; and it will be fully explained in an annotation at another place. 4th. It will be seen that a lamentable deficiency exists in the tables of Boivin and Baudelocque, involving points of the highest practical and statistical magnitude. 5th. The mortality among the French women will strike forcibly the most casual observer, when it is remembered that the forceps are seldom used, and the perforation sacrilegiously interdicted.

But to place our own practice and that of others in their proper light, before the profession, it is necessary that we resort to another tabular history, showing the precise relative frequency of *mortality* and *instrumental labor*. By this table we are willing to be governed, and to award to every man his just position in obstetrical practice.

* Bland says 1 in 44 of his cases were difficult.
† These are transposed by oversight, and should be read so through the whole.

*Table of Mortality and Instrumental Labor.*

| Accoucheurs. | Mortality. | Instrumental. | No. of cases. |
|---|---|---|---|
| Author, | None, | 1 in 67 | 473 |
| Baudelocque, | 1 in 24 | Uncertain | 17,308 |
| Boivin, | No report | 1 in 183 | 20,517 |
| Merriman, | 1 in 210 | 1 in 98 | 2,947 |
| Bland, | 1 in 274 | 1 in 158 | 1,897 |
| Collins, | 1 in 156 | 1 in 114 | 16,654 |
| Ritgen, | No report | 1 in 9 | 103 |
| Kluge, | " | 1 in 15 | 1,111 |
| Carus, | " | 1 in 13 | 2,549 |
| Minden, | " | 1 in 12 | 295 |
| Andree, | " | 1 in 35 | 356 |
| Kustner, | " | 1 in 36 | 368 |
| Boer, | " | 1 in 96 | 9,589 |
| Cusack, | " | 1 in 34 | 1,268 |
| Granville, | " | 1 in 80 | 640 |
| Seibold, | " | 1 in 9 | 340 |
| Voigtel, | " | 1 in 5 | 29 |
| Naegele, | " | 1 in 28 | 1,711 |
| Clarke, | " | 1 in 162 | 10,199 |

The foregoing table exemplifies a remarkable exemption from mortality among lying-in women in this country, and we opine we hazard nothing in saying, that death in child-bed is an event of *uncommon* occurrence in Georgia. That some die, it would be folly to deny; deaths occur in every land, and in all circumstances and conditions of life. The facts in reference to instrumental labor are not so favorable as we could wish, but they can be plausibly and easily explained. 1st. The forceps cases, which are two, occurred both in the same woman, for a lateral pelvic deformity. This cause will continue to exist, and render her amenable to forceps deliveries at every lying-in term. This was irretrievable, and would have occurred to Collins, Baudelocque, Clarke, Boivin or any one else. 2d. The embryotomy and cephalotomy cases supervened under the following circumstances: 1st case. This was a case of impacted head, occurring with a young negress æt. 14; the head was firmly and immovably fixed between the sacrum and pubis; the patient had been in labor many hours, and was rapidly sinking; the child being probably dead, as no pulsation could be detected, I performed cephalotomy; the negress did well, but has never conceived since. Case 2d was a case of arm and shoulder presentation. The woman was a delicate female æt. 38; she was attacked with hemorrhage preceding labor on the day prior to my being called; she was under the care of a *midwife.* I found the arm and shoulder presenting, with a prolapsus of the funis; the throes were violent, and the patient quite exhausted; an attempt at turning was made and abandoned, owing to the death of the child, and the risk incurred of rupturing the uterus. At the suggestion of Dr. Dill, embryotomy was performed; the patient did well. Cases 3d and 4th both occurred in the same individual, and were dependent upon the same causes,—pelvic *narrowness laterally,* and *uterine inertia.* This woman will never give birth to a living child of anything like ordinary dimensions. Every effort was made by myself and others, to rescue these fœtuses from the knife, but without avail: an imperative necessity, essential to the life of the mother, alone induced the operations. Cephalotomy is a painful resort, rendered doubly so where we have any reason to think the child is living, and should never be performed only from the most urgent necessity, and that, too, after due counsel with other practitioners. It is painful enough to perform such an operation, when there is reason to believe the fœtus dead, and I do not envy the feelings or reputation of any man who will plunge his knife into the brain of an innocent babe in utero, without sufficient cause, and after mature, calm and deliberate reflection, in conference with his brethren; yet there are those, who have but little patience, and are anxious to have the opportunity of securing an operative reputation, who will operate without just or sufficient grounds. Happily for women and children, their number is diminutive. It is probable these cases might have been remedied by a resort to the Cesarian operation. An important obstetrical question arises here, as to the validity of that operation. By the Cesarian operation, a risk is incurred of losing both mother and child, while by cephalotomy, *only one is sacrificed.* This is an important item, involving a great moral responsibility. It is the rule in English and American practice to sacrifice the life of the child; in this I fully concur, for reasons which are self evident, and founded upon moral and professional principles. In no event could I be induced to run the risk of sacrificing the life of a mother to save an infant, who in reality has no existence in the external world, but who, whatever may be its fate, must find a resting place in the bosom of the God who gave it. The 5th case was induced by a disproportionate head. This was one of those unavoidable cases which cannot be remedied by manual interference; in consultation, it was resolved to operate, as the only means of saving the mother. This woman had ever had laborious and difficult labor; she was not capacious in her pelvic developements; the proportions of the child were very large; in no event could it have passed the pelvic straits without a diminished head. This closes an account of my cases of instrumental labor. Of the reasons which induced me to operate I shall not speak more definitely than I have, but leave the reader to his own conclusions, with the simple remark, "*that men are fallible, and often differ in their views of right and propriety.*"

*Turning.*—Version in obstetrics is one of those operations which it is easier to talk about than perform. The young obstetrician, who has listened to the declamations of a learned Professor for three or four winters upon the science of midwifery, will find himself quite chagrined at the first introduction he has to a case of several hours standing, requiring turning; ten to one, unless he has great manual dexterity, mixed with a good degree of self-possession and confidence, he will fail. Turning is usually performed by bringing down the feet, but it may be done by the head. In whatever way it is performed, the operator must exercise great caution and care; he must be patient, and remember he is not feeling in a *barrel*, or turning a log of wood about; the least error or undue force, may cost him his reputation for life. I have never performed the operation of version except in arm and side presentations; in one instance the child was saved, the others were lost.

I have usually, where the uterus was violently contracted, administered a full dose of opium before commencing the operation; and after its completion, if the pains were inefficient, I have ordered an infusion of ergot invariably, with the effect of having my anticipations fully realized. It is not the province of this communication to give general directions for the performing of these operations; indeed they can only be learned by careful experience in clinical instruction; I will therefore proceed.

*Convulsions.*—Probably no part of obstetrics is attended with more thrilling interest and intense anxiety than convulsive dystocia. Fortunately, as I have previously remarked, this form of labor seldom occurs among us. The four cases which came under my care, were in three instances in the same person, and of the epileptic variety. The lady is æt. 35; she has had seven births. With her first and every subsequent child she has had puerperal convulsions; she is very subject to premature labor, which is always announced by a convulsion. The convulsions cease as soon as delivery ensues, but prior to that, they resist every therapeutic application for their suspension. I have seen them adopt a strictly tertian type with her, and be partially controlled by quinine; they invariably continue to, and through labor; and I never saw her have one after.

The last attack was in August, '49. I delivered her then of a fœtus at 6 months and 22 days; it is now living, and is a stout, fat child. After these attacks, she has speedy gettings up, and has a return of fine health until her next pregnancy.

The 4th case was a stout, athletic woman, with her first child; the convulsions are of an apoplectic order, unquestionably promoted by an overloaded stomach. This case yielded to *copious venesection, active catharsis*, and *delivery.* As a general rule, not invariable, however, the convulsions cease as soon as the birth of the child takes place, and the physician should be assiduous in his attentions, or the fœtus will be expelled before he is aware of it. These cases usually excite the mind a great deal, and often derange the imagination; causing many painful emotions. I recollect this last case was attacked on Monday, and after convalescence, she experienced a mental horror at the approach of Monday, and in several instances I was sent for to ward off the attack she expected on that day. These are the only cases I have seen prior to parturition, and I know of no physician here who has had a case, although I have made frequent enquiries. It is a protean disease, requiring the promptest attention, and the best of skill.

*Placenta Previa.*—I have met with but two cases of placenta previa; in both of these it was attached partially to the os uteri; they both occurred at the full period. I delivered in both cases the placenta first, and as the pains were violent, the children in both were soon expelled. I am induced to believe that the danger in such cases does not originate from the placental presentation, or it would cease to exist as soon as it was expelled, but it is derived from the exposed orifices of the uterine vessels. It is very clear to my mind that this is correct, from the fact that the placenta is usually detached in the beginning of *labor*, consequently the woman could not suffer from placental hemorrhage, as no affinity exists after that time with her and the child through the placental circulation. Now it is undeniably correct, that in placental presentations, the attachment being about the neck of the uterus, the flooding would be more violent from the exposed orifices, owing to the violent contractions of the body of the womb, producing an expulsion of the fœtus, and a necessary opening of the os uteri. This is a plain but truthful process, and to me fully demonstrates the theory of hemorrhage dependent upon placental presentations. If you deliver the placenta in these cases, the flooding does not cease, but if you deliver both child and placenta it will. We presume no man of any experience will doubt this position; and what does it prove? It surely establishes the point I have taken,—*that placental hemorrhages (as they are called,) depend upon an exposition of the mouths of the uterine vessels, not upon an adherence of the placenta to the os uteri.* What then is the practice? It is *plain, self-evident, and unquestionable; deliver the placenta, promote uterine contraction, and deliver the fœtus as soon as necessary, and with any means your judgment may dictate.*

*Hemorrhage.*—Hemorrhage during pregnancy is not an uncommon occurrence. It is often alarming, requiring the most decided means to stay its progress; when again it is of little importance, requiring nothing more than quietude and the recumbent posture to restore the patient. From the 7th to the 9th month, I have met but two cases; they were mild in their character, and demanded nothing unusual. The emergency of such cases always depends upon the quantity of blood lost and losing, and the ability of the patient to bear it. The points of practice are always to be determined in reference to these facts. I have never had a case of uterine hemorrhage, where I gave ergot prior to delivery.

*Hour Glass Contraction.*—This is dependent upon a circular contraction of the uterus at the point where it exists, and is often a source of considerable annoyance to the accoucheur, particularly if called late. If you are present, the difficulty is usually easily overcome by a gentle introduction of the hand and fingers into the point of constriction, and gently extracting the placenta; if a failure should happen, patience is a fine remedial agent; should all these fail, bleed and give opium. I have never had much difficulty in these cases, but can easily imagine that they can be very annoying. Always bear in mind gentleness is the hand-maid of skill in these cases. Some seem predisposed to hour glass contraction.

*Adherent Placenta.*—This is a perplexing and often difficult complication in obstetrical practice. An adherent placenta is really an untoward event. Fortunately for females, it does not very often exist. Physicians are very frequently called to patients with the belief that they have adhering placentas; this is generally an error of judgment, for in a large majority of cases it is only a retention of the mass. These conditions are widely different and are not to be identified as one in obstetrical practice. Adhesions of the placenta are usually schirrous, cartilaginous, or ossified, and they are to be extracted with great care and gentleness; indeed in all manipulations in the inner womb, "*be gentle,*" should be our motto; *violence is "death."* The hand should be well oiled, and easily introduced, when the placenta should be carefully, cautiously and quietly detached from the uterus. If a portion should remain and cannot be extracted, it will be decomposed and come off; in this case we should use tepid antiseptic enemata and washes daily.

In retention of the placenta, the same caution should be observed. In my own practice I never have a retained placenta. I invariably deliver in 15 minutes after delivery, all things being fair. I have never had any cause to regret the practice, but the experience of every week proves to me its correctness. It is seldom I see a hemorrhage after delivery among my own cases, and I attribute it to the speedy delivery of the after-birth.

*Labor, Duration, Spontaneous Evolution, and anomalous forms.*—Labor is that process by which the child is expelled from the genitals of the woman; it has many varieties, as has been seen, and which it is not necessary to reiterate. Labor may be retarded by several causes, which are laid down in the books; but one of the most common causes of retardation in simple eutocia that I have met with, is the cord being around the neck of the child, and it is much more difficult to remedy than a tyro would suspect. Labor is generally accompanied with pain; but it it not an invariable attendant. I remember to have seen a negress bring forth a fine large child, without the least semblance of pain evident to myself or others; indeed she positively denied having the child until it was exhibited. The duration of labor is a pleasing and interesting question; it is not settled, and will not probably be for many obvious reasons. In a country practice many circumstances conspire to prevent physicians from keeping a record of facts in reference to this subject; it is seldom we are called at the commencement of labor, which accounts at once, in a great degree, for our inability to keep a table; but in my own practice, as far as I have been able to observe, the duration of simple labor does not exceed six hours. The doctrine of spontaneous evolutions in transverse and brachial presentations, was first taught, we believe, by Denman; since which time it has been verified in numerous instances by many accoucheurs; at the present day it is not a problem. I met with a single instance of this evolution; it differed in no respect from other cases of the kind; the child was dead, which is usual in all such cases. I should deem it a rare event for one to be born alive.

In obstetricy the practitioner will meet with many deviations of presentation, which are mentioned by authors; in all such he must be governed by his own superior judgment, and general principles. I have met a case of twins, where one was born in the early part of the day, the other was delayed until the next evening—nearly 36 hours; in this case I was guided alone by general principles; the patient had no *pain or flooding—she was calm and quiet.* I waited patiently; the other child came along in proper order, was sound and healthy; the woman did well. In twins, authors tell us, there is a great proclivity to flooding; I have not found it so; my twin cases do as well as any others. I saw a case in which the cord was torn from the placenta in utero; being present early, I immediately introduced my hand, and brought away the after birth, which had been retained. These errors are remedied with facility, when we are present soon after their occurrence, and the practitioner has the essential ingredients of a skilful tokologist—*self possession and confidence in his ability*; without these, success in every department is equivocal. In two instances labor was impeded by the vulva being effused with serum and blood. The first was punctured, and the case relieved in due season; the second was originally a *tumor*, it became pulsative from an injury. I plunged my lancet into it; the dark grumous and offensive matter it contained was discharged; the labor was soon after completed by the natural efforts. The other cases which came under my inspection, merit no special attention, and I shall not advert to them.

*Ergot.*—Much has been said in justification and denunciation of this drug; by one it is represented as dangerous to the child; by another it is denounced as producing hour glass contractions; and by a third it is proscribed for inefficiency. I have used ergot in almost every form of labor, and I can accord to it none of these properties. In my hands, it has ever been prompt and efficient; I never saw any injury from it in any stage of labor. I believe it excites uterine contractions and promotes dilatation of the *os uteri.* I have never had a case of hemorrhage after giving it prior to delivery. I believe it almost a specific in those cases where there is a tendency to flooding; it should be given 20 or 25 minutes before labor is consummated. The only error it is guilty of, is a predisposition to operate upon the bowels in some women. This can be modified by giving it in conjunction with a little paregoric.

*Pregnancy.*—Hippocrates, Galen, Pliny, La Motte, Haller, Aristotle, Petit, and Levret, contended that pregnancy terminated at the end of nine calendar months, but might be prolonged to ten or fifteen. Now I am not sufficiently versed in gestation to definitively determine this point, but in all the cases I have witnessed in reference to this question, pregnancy has terminated at about the 39th week, plus one day. The quickening period is about the 4th month; five months from this parturition will usually take place. I think the period of gestation is subject to slight changes; it may be a few days over 39 weeks, or a few under it. There is great discrepancy among women in reckoning, and in no instance ought the count of the patient to be strictly relied upon, unless it be with a lady remarkable for her exactness and perception in such things. I remember the case of a lady who brought forth precisely 39 weeks and 2 days from the day of her marriage. I know another instance in which the woman was confined precisely 39 weeks, one day, from the return of her husband who had been gone for four months.

While on the subject of pregnancy, I will remark, that I am inclined to believe in the hereditary influence of labor so far as *quickness, or tediousness of the process is concerned* In a large majority of cases, I have found where women had quick labors, their mothers before them had also, and vice versa. Now I will not assert the rule as invariable, but it is very apt to be the case.

I have now gone through what I fully intended when I began—*a synoptical account of my obstetrical experience.* I have extended the paper much farther than I at first intended to do. I hope, however, its unforeseen prolixity will not be at all detrimental. It is possible in some parts of its serpentine course, I may have committed some errors, if so, they are unintentional and will be pleasurably rectified. I have not thought proper to pursue a strictly systematic course in treating of the various topics involved, for the reason thatthe paper was intended as a practical effort, with no didactic intention, but only as my own experience. In conclusion, I will make a small tabular view of facts and observations in reference to general items.

*Miscellaneous Table.*

Duration of labor, 6 hours.
Duration of pregnancy, 39 weeks, 1 day.
1 Case spontaneous evolution.
1 Case 36 hours between births.
1 Case without pain in labor.
1 Case child with teeth.
1 Case imperforate anus.
1 Case hydatids, (*not mentioned in tables.*)
4 Cases still born, *restored.*
2 Forceps cases, both children living.
In a majority of all the cases, males predominated to a small extent.

